# Whole-Genome Sequence of Drug-Resistant Mycobacterium tuberculosis Strain S7, Isolated from a Patient with Pulmonary Tuberculosis

**DOI:** 10.1128/MRA.01567-19

**Published:** 2020-04-23

**Authors:** Aparna Meher, Hritusree Guha, Raghuveer Varma Pemmadi, Subia Akram, Hossain M. Faruquee, Rakesh Arya, Hiren Ghosh, Chaitali Nikam, Sourav Saha, Ranjit Das, Arunabha Dasgupta, Bidhan Goswami, Dinesh Gupta, Anjan Das, Ranjan Kumar Nanda

**Affiliations:** a Translational Health Group, International Centre for Genetic Engineering and Biotechnology, New Delhi, India; b Department of Respiratory Medicine, Agartala Government Medical College, Kunjaban, Agartala, India; c Department of Pharmaceutical Chemistry, Raghavendra Institute of Pharmaceutical Education and Research-Autonomous, Anantapur, Andhra Pradesh, India; d Department of Biotechnology and Genetic Engineering, Islamic University, Kushtia, Bangladesh; e University Medical Center Groningen, Groningen, The Netherlands; f Thyrocare Technologies Ltd., Navi Mumbai, India; g Department of Medicine, Agartala Government Medical College, Kunjaban, Agartala, India; h Department of Microbiology, Agartala Government Medical College, Kunjaban, Agartala, India; i Translational Bioinformatics Group, International Centre for Genetic Engineering and Biotechnology, New Delhi, India; University of Maryland School of Medicine

## Abstract

Over the past decades, drug-resistant Mycobacterium tuberculosis strains have presented a significant challenge, with inadequate diagnosis of tuberculosis (TB) cases. Here, we report the draft whole-genome sequence of drug-resistant M. tuberculosis strain S7, which was isolated from a patient from Tripura, India, who was diagnosed with pulmonary TB.

## ANNOUNCEMENT

The worldwide emergence and spread of tuberculosis (TB) cases resistant to the available anti-TB drugs pose serious public health threats. In particular, India accounts for the greatest number of TB cases, nearly one-fourth of the total global TB burden, with an equivalent share of drug-resistant TB cases (1, 2).

Here, we report the whole-genome sequence of Mycobacterium tuberculosis strain S7, which was isolated from a patient from Tripura, India, who was diagnosed with pulmonary TB. The sputum sample was processed and cultured at 37°C in a mycobacterial growth indicator tube (MGIT) with polymyxin B-amphotericin-nalidixic acid-trimethoprim-azlocillin (PANTA) supplement. The drug susceptibility test (DST) results revealed resistance to the anti-TB drugs isoniazid (0.1 μg/ml) and clofazimine (0.5 μg/ml).

Study protocols adopted in this study were approved by the human ethics committees of the International Centre for Genetic Engineering and Biotechnology (New Delhi, India) (protocol ICGEB/IEC/2017/07) and Agartala Government Medical College (Agartala, India) (protocol F.4[6-9]/AGMC/Academic/IEC Committee/2015/8965, dated 25 April 2018). After receiving an informed signed consent form to participate in this study, subjects were recruited at Agartala Government Medical College.

An aliquot from the MGIT was transferred into 7H9 medium with Middlebrook oleic acid-albumin-dextrose-catalase (OADC) growth supplement (10%) and grown at 37°C in a shaking incubator. The genomic DNA was extracted from the exponentially growing M. tuberculosis S7 culture (optical density at 600 nm, 1.0) following the protocol reported by Warren et al. (3). Sequencing was performed on a HiSeq 4000 system (Illumina, Inc., San Diego, CA, USA) using the TruSeq SBS v3 reagent kit (2 × 150 bp) for library preparation. The quality of Illumina raw reads was checked using fastp v0.20.0 (4), which indicated that the sequencing coverage was 100×. After filtering, a total of 24,624,392 reads, with 93.44% of bases with a quality score above Q30, were found. The *de novo* genome assembly was performed using SPAdes v3.13.2 (5), and the assembly quality was assessed using QUAST v5.0.2 (6). The assembly comprised 206 contigs (81 contigs of ≥500 bp), with a total length of 4,401,606 bp, an *N*_50_ value of 129,971 bp, and an average GC content of 65.6%. Raw assembled contigs were aligned against the reference genome H37Rv (GenBank accession number NC_000962.3) using Mauve version snapshot 2015-02-13 (7), resulting in a total of 57 locally colinear blocks, 1,089 single-nucleotide polymorphisms, and 179 gaps in the assembly. Genome annotation was performed using Prokka v1.14.0, with default parameter settings (8). A total of 4,044 coding sequences, 53 tRNAs, and 3 rRNAs were predicted. Default parameters were used for all software unless otherwise noted.

Species identification and drug resistance prediction were performed with Mykrobe v0.7.0 (9), and the strain was identified to be of European-American lineage. A predicted mutation from C to T in the *fabG1* gene, at position 1673425 of the reference genome, was annotated for isoniazid resistance, which confirms our DST results. ResFinder v3.2 (10) predicted two antimicrobial resistance genes, namely, *aac(2′)-Ic* and *erm*(37), which are involved in aminoglycoside and macrolide resistance, respectively. Additionally, unknown mutations were identified in *gyrA* (p.E21Q, p.S95T, and p.G668D) and *ethR* (p.A9T) genes in M. tuberculosis S7. None of the aforementioned tools used for drug resistance prediction identified genes responsible for clofazimine resistance. The availability of high-quality genome data for the M. tuberculosis S7 strain will serve as a valuable resource for comparative studies on the epidemiology of globally emerging drug-resistant TB.

### Data availability.

The raw reads of the whole-genome sequence of M. tuberculosis S7 were submitted to the SRA under accession number SRR10695721. The GenBank accession number is WUJD00000000.

## References

[1] Corbett EL, Watt CJ, Walker N, Maher D, Williams BG, Raviglione MC, Dye C 2003 The growing burden of tuberculosis: global trends and interactions with the HIV epidemic. Arch Intern Med 163:1009–1021. doi:10.1001/archinte.163.9.1009. 12742798

[2] Arinaminpathy N, Batra D, Khaparde S, Vualnam T, Maheshwari N, Sharma L, Nair SA, Dewan P 2016 The number of privately treated tuberculosis cases in India: an estimation from drug sales data. Lancet Infect Dis 16:1255–1260. doi:10.1016/S1473-3099(16)30259-6. 27568356 PMC5067370

[3] Warren R, Kock M, Engelke E, Myburgh R, Pittius NG, Victor T, Helden P 2006 Safe *Mycobacterium tuberculosis* DNA extraction method that does not compromise integrity. J Clin Microbiol 44:254–256. doi:10.1128/JCM.44.1.254-256.2006. 16390984 PMC1351970

[4] Chen S, Zhou Y, Chen Y, Gu J 2018 fastp: an ultra-fast all-in-one FASTQ preprocessor. Bioinformatics 34:i884–i890. doi:10.1093/bioinformatics/bty560. 30423086 PMC6129281

[5] Bankevich A, Nurk S, Antipov D, Gurevich AA, Dvorkin M, Kulikov AS, Lesin VM, Nikolenko SI, Pham S, Prjibelski AD, Pyshkin AV, Sirotkin AV, Vyahhi N, Tesler G, Alekseyev MA, Pevzner PA 2012 SPAdes: a new genome assembly algorithm and its applications to single-cell sequencing. J Comput Biol 19:455–477. doi:10.1089/cmb.2012.0021. 22506599 PMC3342519

[6] Gurevich A, Saveliev V, Vyahhi N, Tesler G 2013 QUAST: quality assessment tool for genome assemblies. Bioinformatics 29:1072–1075. doi:10.1093/bioinformatics/btt086. 23422339 PMC3624806

[7] Darling AC, Mau B, Blattner FR, Perna NT 2004 Mauve: multiple alignment of conserved genomic sequence with rearrangements. Genome Res 14:1394–1403. doi:10.1101/gr.2289704. 15231754 PMC442156

[8] Seemann T 2014 Prokka: rapid prokaryotic genome annotation. Bioinformatics 30:2068–2069. doi:10.1093/bioinformatics/btu153. 24642063

[9] Walker TM, Kohl TA, Omar SV, Hedge J, Del Ojo Elias C, Bradley P, Iqbal Z, Feuerriegel S, Niehaus KE, Wilson DJ, Clifton DA, Kapatai G, Ip CLC, Bowden R, Drobniewski FA, Allix-Béguec C, Gaudin C, Parkhill J, Diel R, Supply P, Crook DW, Smith EG, Walker AS, Ismail N, Niemann S, Peto TEA, Modernizing Medical Microbiology (MMM) Informatics Group 2015 Whole-genome sequencing for prediction of *Mycobacterium tuberculosis* drug susceptibility and resistance: a retrospective cohort study. Lancet Infect Dis 15:1193–1202. doi:10.1016/S1473-3099(15)00062-6. 26116186 PMC4579482

[10] Zankari E, Hasman H, Cosentino S, Vestergaard M, Rasmussen S, Lund O, Aarestrup FM, Larsen MV 2012 Identification of acquired antimicrobial resistance genes. J Antimicrob Chemother 67:2640–2644. doi:10.1093/jac/dks261. 22782487 PMC3468078

